# Spiritual Well-Being and Treatment Adherence in Patients with Type 2 Diabetes in Turkey

**DOI:** 10.1007/s10943-025-02271-0

**Published:** 2025-02-20

**Authors:** Seçil Erden Melikoğlu, Berna Köktürk Dalcali, Esra Güngörmüş, Hatice Kaya

**Affiliations:** 1 Department of Fundamentals of Nursing, Florence Nightingale Faculty of Nursing, Istanbul University-Cerrahpaşa, Abide-i Hurriyet Street, 34381 Sisli, Istanbul, Turkey; 2 Faculty of Health Sciences, Bandırma Onyedi Eylül University, Bandırma, Balıkesir, Turkey; 3 Cerrahpaşa Medical School, Istanbul University-Cerrahpaşa, Istanbul, Turkey

**Keywords:** Type 2 diabetes, Treatment adherence, Spiritual well-being, Nursing care

## Abstract

This study aimed to identify the correlation between treatment adherence and spiritual well-being in patients with Type 2 Diabetes. A descriptive and correlational design was used. The study consisted of 203 patients. The data were collected through a patient information form, The Scale for Compliance to the Treatment in Type 2 Diabetes Mellitus and the FACIT-Sp-12 and analyzed by using descriptive and Pearson’s Correlation analysis. A correlation was found between patients treatment adherence and their peace, faith, and meaning sub-dimensions of the FACIT Sp-12. This study shows a need for interventions for patients with Type 2 diabetes to increase their spiritual well-being and, therefore, their treatment adherence. For this reason, individuals’ needs should be met with a holistic approach by integrating spiritual care into nursing care practices.

## Introduction

One of the important chronic diseases increasing the risk of morbidity and mortality with its acute and chronic complications, requiring constant treatment and care, and causing a high cost of care is diabetes mellitus (DM) (International Diabetes Federation (IDF), 2019; Turkish Society of Endocrinology and Metabolism (TEMD), 2020). Although the prevalence of diabetes varies from society to society, the common point is that its prevalence is increasingly growing globally and that it has become a growing public health problem by affecting more and more people every other day (Elsous et al., 2017). As a result of decreased physical activity and changes in nutritional habits, the prevalence of particularly Type 2 diabetes is increasingly growing throughout the world, including both developed and developing countries (IDF, 2019; TEMD, 2020). The 2019 data of the IDF shows that the current population of diabetics is 463 million people globally and that it is predicted to reach 700 million people by 2045.

In addition to the findings related to diabetes, the physical, mental, psychological, and social life of the individual is affected by the distress caused by the treatment, anxiety about the future, and fears about body image (Bahar & Tanrıverdi, 2017). Along with these psychosocial changes, glycemic control and adherence with treatment are also affected (Jafari et al., 2014; Kes & Gökdoğan, 2020). Patients with diabetes need lifestyle changes and self-care to take the disease under control. It is emphasized that the most important barrier that complicates diabetes management is related to non-adherence to treatment (Javanmardifard et al., 2020; Krass et al., 2015). Several factors affect treatment adherence in patients with Type 2 diabetes. Of these factors, spirituality is stated to be an important one in patients' treatment adherence and coping effectively (Baby & Khan, 2016; Movahed et al., 2020).

Spirituality can be used as a coping mechanism that can help the individual cope with the disease and adhere to treatment by providing confidence and hope (Heidarzadeh & Aghamohammadi, 2017). Individuals can maintain their self-respect and have an understanding of themselves thanks to spirituality. In addition, it provides strength, relief, hope, and peace for people so that they can cope with their problems, and it is also useful in terms of many issues, including facilitating individuals acceptance of their illness, lowering depression and stress, increasing their quality of life, and enabling them to assume social responsibility (Erişen & Sivrikaya, 2017). Spirituality has a significant role in controlling pain during treatment, reducing anxiety, adapting to illness, and disease management, as well as preventing diseases (Baby & Khan, 2016; Rafferty et al., 2015). There is some research into the correlation between treatment adherence and spiritual well-being in individuals with diabetes in different countries, but no research findings on this subject in Turkey have been found. In this context, this research was planned to examine the correlation between treatment adherence and spiritual well-being in individuals with Type 2 Diabetes. Accordingly, the answers to the following questions were sought in this study:

1. What is the level of spiritual well-being in patients with Type 2 Diabetes?
2. What is the level of treatment adherence in patients with Type 2 Diabetes?
3. Is there any difference between treatment adherence and socio-demographic and disease characteristics in patients with Type 2 Diabetes?
4. Is there a correlation between the spiritual well-being of patients with Type 2 Diabetes and their treatment adherence?

## Methods

### Design

This study was carried out in a descriptive and correlational design to examine the correlation between treatment adherence and spiritual well-being in individuals with Type 2 Diabetes.

### Sampling and Setting

Individuals with Type 2 Diabetes who visited the diabetes outpatient clinic of a university hospital in Turkey from April 2021 to September 2021 made up the study population. The study sample included individuals meeting the sampling criteria and agreeing to join the study between these dates. The number of individuals to be recruited was calculated via a power analysis on the G*Power (v3.1.9) software. In general, 1-β (β = probability of type II error) is used to show the power of a study, and a study is expected to have 80% power. Based on the estimated medium effect size (ρ = 0.3) in the correlation analyses to be performed, the sample size of the study was calculated as at least 82 individuals to obtain 80% power at α = 0.05 level (Cohen, 1992). Eventually, the study was conducted with 203 patients who had had Type 2 diabetes for at least one year, were aged 18 or older, and agreed to join the study voluntarily.

### Data Collection Tools

A Patient Information Form, The Scale for Compliance to the Treatment in Type 2 Diabetes Mellitus, and the FACIT-Sp-12 (V.4) (Spiritual Well-being Scale) were used to collect the study data.

### The Patient Information Form

This form was prepared following a review of the literature and consisted of questions about disease-related and socio-demographic characteristics of participants with Type 2 diabetes (Şahin et al., 2021; Turen et al., 2021).

### The FACIT-Sp-12 (Version 4)

This is a spiritual well-being scale which was created by Peterman et al. (2002) to determine the level of spiritual well-being in individuals with cancer and other chronic diseases. Aktürk et al. (2017) conducted the reliability and validity study of the scale in Turkey. The scale had 12 items, each of which is scored on a 5-point Likert-type evaluation structure. It consists of three subscales, namely peace, meaning, and faith. The peace subscale is used to evaluate inner peace, the meaning subscale helps the individual to evaluate his/her inner harmony, and the faith subscale helps the individual to evaluate the state of obtaining strength and comfort in his/her religious beliefs. Items on the scale are given scores from 0 to 4 points, and total scores on the scale range from 0 to 48. High scores on the scale mean high levels of spiritual well-being. Cronbach’s alpha of the scale in the Turkish validity and reliability study was 0.87 (Aktürk et al., 2017). The Cronbach’s alpha value in this study was determined as 0.82.

### The Scale for Compliance to the Treatment in Type 2 Diabetes Mellitus

Demirtaş and Akbayrak (2014) created this scale to evaluate patients’ treatment adherence to Type 2 DM. The scale, which can be applied to patients who have had Type 2 diabetes for at least 1 year, has 30 items, each of which are scored on a 5-point Likert-type scale. Of the items, 13 are positive and 17 are negative. Total scores on the scale vary from 30 to 150. The interpretation of the scores is as follows: 30–54 points indicate "good treatment adherence"; 55–125 points indicate "medium treatment adherence"; 126–150 points indicate "poor treatment adherence." Cronbach’s alpha value of the scale was found to be 0.77 (Demirtaş & Akbayrak, 2014). The Cronbach’s alpha value of the scale in this study was found to be 0.83.

### Data Collection

Before data collection was initiated, the approval of the relevant institution and ethics committee was obtained. The researcher explained the aim of the study to the patients in one-to-one interviews. First, participants submitted informed consent, and then data collection process was initiated. The face-to-face interview method was utilized to collect study data from patients who agreed to participate in the study.

## Data Analysis

Study data were analyzed on R V.2.15.3 software package (R Core Team, 2013). For the evaluation of study data, descriptive statistical methods were utilized. The Shapiro–Wilk test, skewness and kurtosis values and graphical examinations were used to test the normality of the quantitative data. Independent samples t-test was utilized for the paired evaluation of variables with a normal distribution. The Mann–Whitney U test was utilized for the paired analysis of variables with a non-normal distribution. The multiple group assessment of the variables that did not show normal distribution was performed via the Kruskal–Wallis test, and when a significant result was found, the source of the significance was identified via the Dunn-Bonferroni test. The level of correlation between quantitative variables was identified via Pearson correlation analysis. Cronbach's alpha value was utilized to determine the levels of internal consistency. Statistical significance was taken as *p* < 0.05.

### Ethical Aspects of the Study

The study was approved by the Non-Interventional Clinical Research Ethics Committee of a university (date: 21.02.2020 issue: 2020–04). The written institutional permission of the hospital where the research would be conducted was obtained. In addition, the written permission of the authors who carried out the Turkish reliability and validity studies of the scales that were employed in the study was obtained. Informed consent of the patients who agreed to join the study was obtained in accordance with the principle of voluntariness. The principles of Helsinki Declaration were followed in each stage of the research.

## Results

Participants’ mean age was 56.38 ± 9.66 years, 82.8% were married, 60.6% were female, 61.6% had primary school education, 61.6% had equal income and expenses, and 70.0% were unemployed (Table 1).

**Table 1:** Distribution of patients with Type 2 Diabetes by descriptive characteristics (N = 203)

Descriptive characteristics	Min.-Max	Mean ± SD
Mean age (year)	31–84	56.38 ± 9.66
	N	%
*Gender*		
Female	123	60.6
Male	80	39.4
*Marital status*		
Married	168	82.8
Single	35	17.2
*Level of education*		
Not literate	12	5.9
Primary education	125	61.6
High school	33	16.3
University	33	16.3
*Working status*		
Non-working	142	70.0
Full-time	53	26.1
Part-time	3	1.5
Flexible	5	2.5
*Income level*		
Income < expenses	71	35.0
Income = expenses	125	61.6
Income > expenses	7	3.4

The mean duration of diabetes diagnosis of patients in the study was 124.65 ± 91.44 months, and the mean duration of diabetes treatment was 118.81 ± 91.69 months. Also, 46.3% were using oral antidiabetic drugs (OAD) as a treatment, 45.8% had received diabetes education, and 7.9% were hospitalized due to diabetes (Table 2).

**Table 2:** Distribution of patients with Type 2 Diabetes by disease-related characteristics (N = 203)

Disease-related characteristics	Min.-Max	Mean ± SD
Duration of diabetes diagnosis (month)	12–480	124.65 ± 91.44
Duration of diabetes treatment (month)	1–480	118.81 ± 91.69
	N	%
*Type of diabetes treatment*		
OAD	94	46.3
OAD + Diet + Exercise	14	6.9
Insulin	15	7.4
OAD + Insulin	73	36.0
OAD + Insulin + Exercise	7	3.4
*Status of having received diabetes education*		
Yes	93	45.8
No	110	54.2
*Hospitalization due to diabetes or complications in the last year*		
Yes	16	7.9
No	187	92.1

OAD: Oral antidiabetic drug

Participants’ mean score on the total FACIT Sp-12 scale was found to be 30.37 ± 6.99. The mean scores on the sub-dimensions were determined as 11.30 ± 2.58 for the meaning sub-dimension, 9.14 ± 2.96 for the peace sub-dimension, and 9.93 ± 3.00 for the faith sub-dimension. Patients’ mean score on the Scale for Compliance to the Treatment in Type 2 DM was found to be 84.58 ± 13.31 (Table 3).

**Table 3:** Distribution of patients’ scores on the total spiritual well-being scale and its sub-dimensions and the scale for compliance to the treatment in Type 2 Diabetes

The scales	Min.-Max	Mean ± SD
FACIT-Sp-12 Scale		
Meaning	4–16	11.30 ± 2.58
Peace	2–16	9.14 ± 2.96
Faith	2–16	9.93 ± 3.00
Total scale score	11–48	30.37 ± 6.99
The scale for compliance to the treatment in Type 2 Diabetes Mellitus	44–110	84.58 ± 13.31

It was observed that patients who used OAD in the treatment of diabetes and followed a diet and exercise had better treatment adherence than those using OAD, insulin, and insulin and OAD. It was determined that patients who had already received diabetes education had better treatment adherence than patients who had not (Table 4). No significant difference was identified between patients' treatment adherence and gender, marital status, income status, educational status, and employment status (*p* > 0.05). Also, no significant relationship was identified between patients’ treatment adherence and their age, the duration of diabetes diagnosis, and the duration of treatment (*p* > 0.05).

**Table 4:** Comparison of patients’ descriptive characteristics and their mean scores on the total the scale for compliance to the treatment in Type 2 Diabetes

Variables	Patient adherence to type 2 Diabetes mellitus treatment scale	
	N	Median (Min–Max)
*Diabetes treatment*		
OAD	94	85 (77. 94)
OAD + Diet + Exercise	14	69.5 (57. 78)
Insulin	15	86 (82. 92)
OAD + Insulin	73	92 (83. 97)
OAD + Insulin + Exercise	7	63 (55. 86)
^b^χ^2^		31.032
P		< 0.001*
Status of having received diabetes education	N	Mean ± SD
No	110	86.7 ± 12.37
Yes	93	82.79 ± 13.85
^a^t		− 2.103
p		0.037*

r = Pearson correlation coefficient ^a^Independent samples t-test ^b^Kruskal-Wallis test. *p < 0.05 OAD: Oral antidiabetic drug

A correlation was identified between the mean scores of patients with Type 2 Diabetes in the study on the peace (r = − 0.608, *p* < 0.001), faith (r = -0.372, *p* < 0.001), and meaning (r = − 0.493, *p* < 0.001) sub-dimensions of the FACIT Sp-12 scale and their mean score on the total Scale for Compliance to the Treatment in Type 2 DM (r = − 0.599, *p* < 0.001) (Table 5).

**Table 5:** The correlation between patients’ mean scores on the sub-dimensions of the spiritual well-being scale and the total scale for compliance to the treatment in Type 2 Diabetes Mellitus

	The scale for compliance to the treatment in Type 2 Diabetes Mellitus	
^**1**^FACIT-Sp-12 Meaning	r	− 0.493
	*p*	< 0.001*
^2^FACIT-Sp-12 Peace	r	− 0.608
	*p*	< 0.001*
^**3**^FACIT-Sp-12 Faith	r	− 0.372
	*P*	< 0.001*

r = Pearson correlation coefficient *p < 0.05

## Discussion

Diabetes has become a social and global health problem today and brings about many adaptation problems in patients. Treatment adherence is an important issue, particularly in taking the disease under control. It is known that spiritual well-being is important in individuals with diabetes in terms of individual self-management and health promotion behaviors as in other chronic diseases (Choi & Hastings, 2019; Onyishi et al., 2021).

In the study, the spiritual well-being level of patients who had Type 2 diabetes was above the average. Spiritual well-being is an internal energy that adds meaning to the lives of individuals, helps them to control stress and cope with physical and psychological pain, and provides effective management of the disease (Movahed et al., 2020). In Turkey, some studies investigating spiritual well-being in different patient groups reported moderate well-being (Köktürk Dalcalı et al., 2021), while others reported it as high (Yılmaz & Cengiz, 2020). Research in the literature into determining the spiritual well-being of diabetic patients has shown varying degrees. For example, Faghani et al. (2018) and Durmuş et al. (2022) reported that it was above average, while Javanmardifard et al. (2020) reported an average level. However, Jafari et al. (2014) reported the levels of spiritual well-being as low. Sitepu and Pohan (2020) reported that 92.3% of patients with diabetes mellitus in their study had high levels of spiritual well-being. It has been reported that the high levels of spiritual well-being in Muslim societies may be related to the faith sub-dimension, but in the study of Jafari et al. (2014), the scores of the patients on the meaning sub-dimension were higher. It is thought these differences can be seen the studies were conducted with diabetic patients with different socio-cultural, religious characteristics and individuals have different personal belief systems.

According to our findings, the treatment adherence of patients who had Type 2 diabetes in our study was moderate. Özonuk and Yılmaz (2019), in their study conducted with patients who had Type 2 Diabetes, determined the treatment adherence of patients as moderate. Similarly, Turen et al. (2021), Şahin et al. (2021), and Eşki and Baysal (2022) also found that the treatment adherence of patients who had Type 2 diabetes was moderate. Eşer et al. (2018) found that 97.4% of patients who had Type 2 Diabetes had a moderate level of treatment adherence. Özkaptan et al. (2019), too, determined that 98.3% of patients who had Type 2 Diabetes adhered to treatment at a moderate level. However, in the study of Kes and Gökdoğan (2020) conducted with patients who had Type 2 Diabetes, patients' treatment adherence was found to be poor. In their study carried out in Tehran, Pazokian et al. (2020) found that patients who had Type 2 Diabetes had a low treatment adherence level. Ishaq et al. (2021), in their study conducted in Pakistan, concluded that the treatment adherence of patients who had Type 2 Diabetes was moderate. Some studies have shown that there is a difference between the treatment adherence levels of patients who have Type 2 Diabetes. It is thought these differences can be seen based on the health care service delivery, the socio-economic status of individuals, their perceptions and beliefs about the disease, and the factors associated with the disease process, as well as different scales used to assess treatment adherence. Krass et al. (2015) reported in their meta-analysis that drug compliance of patients with diabetes was between 38.5 and 93%, and it was emphasized that adherence to the treatment of Type 2 diabetes was an ongoing problem in different country samples. The study results revealed that compliance with the treatment of diabetes was not at the desired level and that new strategies on how to maintain effective diabetes management needed to be put into practice.

According to the study findings, no significant difference was identified between patients' treatment adherence and their educational status, gender, marital status, income status, and employment. Similarly, Eşer et al. (2018) found no significant difference between treatment adherence and marital status, gender, income status, education level, and employment status. Şahin et al. (2021) found no significant difference between the treatment adherence of patients with diabetes and their educational status, gender, and income level. Eşki and Baysal (2022) determined no significant difference between treatment adherence and educational status, gender, and marital status. Unlike these studies in the literature, which indicated no correlation between patients’ education level and their treatment adherence, Özkaptan et al. (2019), Gholamaliei et al. (2016), and Javanmardifard et al. (2020) reported that as patients’ education level increased, their treatment adherence increased, as well. The results of the study indicated that there was no significant correlation between treatment adherence and the duration of diabetes diagnosis. When the literature was examined, it was observed that there was no significant relationship between the duration of diabetes diagnosis and treatment adherence (Ekşi & Baysal, 2022; Eşer et al., 2018; Özkaptan et al., 2019). According to the findings of the present study, it was determined that patients who used OAD in the treatment of diabetes and followed diet and exercise showed better treatment adherence than patients who used OAD and insulin therapy. Özkaptan et al. (2019) also found that patients who used OAD and insulin together had lower levels of treatment adherence than patients who used only OAD. In the current study, patients who had already received diabetes education had better treatment adherence. In their study on patients who had Type 2 Diabetes, Özonuk and Yılmaz (2019) determined that individuals who had already received education on diabetes had better treatment adherence than those who had not. Şahin et al. (2021) determined no significant correlation between having received diabetes education and treatment adherence. It is thought these differences can be seen the studies were conducted with diabetic patients with different socio-cultural and educational levels.

The results of the study indicated that there was a correlation between patients' spiritual well-being levels and their treatment adherence and that an increase in patients’ spiritual well-being level meant an increase in their treatment adherence. Similarly, Alvarez et al. (2014) reported a significant correlation between patients' treatment adherence and their spiritual well-being. Jafari et al. (2014) found that individuals who could take their diabetes under control had a higher level of spiritual well-being than individuals who could not. In their qualitative study with patients with diabetes, Namageyo-Funa et al. (2015) reported that spirituality helped patients to manage diabetes. Darvyri et al. (2018) emphasized that spirituality had an important effect on Type 2 diabetes management as it helped achieve better glycemic control. These results show the need for spiritual care practices during the provision of care for patients who have diabetes. In contrast, Javanmardifard et al. (2020) found that the level of patients’ spiritual well-being negatively affected their treatment adherence. They explained this situation by stating that patients who had high levels of spiritual well-being believed that nothing would happen outside of God's will and that God would protect them. Duke and Wigley (2016) obtained results that were similar to those of Javanmardifard et al. (2020) and they stated spiritual well-being and treatment adherence were negatively correlated. Shahdadi et al. (2015) showed that there was no correlation between glycemic index control and spiritual well-being. It is thought that different results obtained from the studies mentioned might have stemmed from the fact that these studies were conducted with patients with diabetes with different socio-cultural and religious characteristics.

### Limitation

The study sample was made up of only patients who were followed up at a university hospital diabetes clinic. Since there is no other measurement tool that assesses spiritual well-being which has been validated in Turkish, the FACIT-Sp-12 scale developed by Peterman et al. (2002) and adapted to Turkish by Aktürk et al. (2017) was used in this study. However, Koeing and Carey (2024) reported in their study that the peace and meaning sub-dimension is an indicator of mental health, not spirituality, and the faith sub-dimension indicates spiritual well-being. In another study by Koeing and Carey (2025), notes that the association of total scores on such contaminated measures with health outcomes should not be examined. In line with these studies, the relationship between adherence to diabetes treatment and the total score of the spiritual well-being scale was not examined. At the same time, the results regarding the relationship between the scores obtained from the peace and meaning sub-dimensions and adherence to diabetes treatment have limitations. The relationship between spiritual well-being and adherence to diabetes treatment should be evaluated in the context of the faith sub-dimension.

## Conclusion

It was determined that the level of spiritual well-being in patients who had Type 2 diabetes was above the average and that their treatment adherence was at a moderate level. There was a relationship between the spiritual well-being and treatment adherence of patients. It was concluded that as patients’ spiritual well-being levels increased, their treatment adherence would also increase. In this context, it is important to support spiritual care practices to sustain patients' adherence to treatment.

According to the results of the research, it is understood that there is a need for interventions to increase the spiritual well-being levels of patients who have Type 2 diabetes and their treatment adherence. Spiritual care has a significant part to play in improving treatment adherence in patients who have diabetes. Therefore, individuals’ needs should be met with a holistic approach by integrating spiritual care into nursing care practices. In this context, it is recommended to organize education programs to increase nurses' awareness and practice skills regarding spiritual care and to include spiritual care practices, which are important components of the holistic approach, in in-service training programs.
